# A Study on the Relationship between Postural Control and Pain-Related Clinical Outcomes in Patients with Chronic Nonspecific Low Back Pain

**DOI:** 10.1155/2021/9054152

**Published:** 2021-11-02

**Authors:** Qiuhua Yu, Yunxia Huo, Min Chen, Zhou Zhang, Zhicheng Li, Haizhen Luo, Zhenwen Liang, Chuhuai Wang, Wai Leung Ambrose Lo

**Affiliations:** ^1^Department of Rehabilitation Medicine, The First Affiliated Hospital, Sun Yat-Sen University, Guangzhou, China; ^2^Department of Rehabilitation Medicine, First Affiliated Hospital of Xi'an Jiaotong University, Xi'an, China; ^3^Biomedical Research Institute, Shenzhen-Peking University-The Hong Kong University of Science and Technology Medical Center, Shenzhen, China; ^4^Department of Radiology, The First Affiliated Hospital, Sun Yat-Sen University, Guangzhou, China; ^5^Department of Rehabilitation Medicine, The First Affiliated Hospital of Jinan University, Guangzhou, China; ^6^Guangdong Engineering and Technology Research Centre for Rehabilitation Medicine and Translation, The First Affiliated Hospital, Sun Yat-Sen University, Guangzhou, China

## Abstract

**Objectives:**

To explore the relationship between postural control and pain-related clinical outcomes in patients with chronic nonspecific low back pain (cNLBP).

**Methods:**

Participants with cNLBP and healthy individuals were recruited. Muscle activities were recorded during internal and external perturbation tasks. Postural control capacity was assessed by muscle onset time and integrals of electromyography (iEMGs) of postural muscles during the phases of anticipatory postural adjustments (APAs) and compensatory postural adjustments (CPAs). Correlation analysis was employed to investigate the relationship between postural control capacity, pain, and disability.

**Results:**

Twenty-seven patients with cNLBP and 27 healthy participants were recruited. Gastrocnemius (GA) muscle onset time was earlier in the cNLBP group than in the control group in the internal perturbation task. The onset time of GA and erector spinae (ES) of the cNLBP group was later than that of the controls in the external perturbation task. Disability level moderately correlated with the iEMGs of rectus abdominis (RA), GA, and external oblique (EO) during APAs. Pain score moderately correlated with the iEMGs of RA, EO, and ES during CPAs of perturbation tasks.

**Conclusion:**

cNLBP participants had altered muscle activation strategy to maintain postural stability in response to perturbation. This study further discovered that pain-related disabilities of cNLBP participants were likely related to the APAs capacity, whereas the pain intensity may relate to the CPAs capacity. Pain and disability may therefore be related to the control process of the posture-related muscles.

## 1. Introduction

Chronic low back pain (cLBP) is a common health problem that causes heavy social and economic burden [1]. Among 76% of people with cLBP were diagnosed with chronic nonspecific low back pain (cNLBP). cNLBP is characterized by pain sensation that appears between the 12th rib and the hip with or without leg pain [1]. The exact mechanism that contributes to cNLBP remains unknown, and there continues to be a debate surrounding which biological marker may contribute to the reoccurrence of pain.

Among numerous proposed theories, altered motor control is commonly reported as a contributing factor to the persistence of pain [2]. Altered motor control often refers to the activation timing of the core muscles, e.g., transverse abdominus, in response to a postural perturbation task. This resulted in suboptimal load placed on the passive tissues of the spine during movement and impaired balance function, which contribute to symptoms occurrence [3, 4]. The ability to maintain balance is related to postural control adjustments, which could be broadly classified as anticipatory postural adjustments (APAs) and compensatory postural adjustments (CPAs) [5, 6]. APA is the muscle adjustment prior to the onset of perturbation, whereas CPA is the adjustment of postural muscle activity occurred after the onset of perturbation. Therefore, APA is comparable to feed-forward control (FFC), which is a top-down control elicited by motor intention or external input information [7]. On the contrary, CPA is comparable to the feedback control, which is a bottom-up control in response to imperative perturbation [7].

There is a body of literature that reported APAs dysfunction in patients with cNLBP [2, 8, 9]. The findings of these studies showed that the onset time of the postural muscles in patients with cNLBP was significantly delayed before the perturbation elicited by either a sudden change of trunk load or the rapid arm lifting test. Hedayati et al. found that the participants with LBP exhibited less variability in the contraction timing ratio of APAs of the transverse abdominis and internal oblique muscles when compared with healthy individuals [8]. The reduction in the time variability of APAs is indicative of an unstable system that has reduced adaptability to internal or external perturbations. The potential reason for less variability in timing of APAs was that increasing trunk stiffness and cocontraction is to reserve spinal stability for cLBP patients. Besides APAs, the cNLBP patients also showed CPAs dysfunction of trunk muscles [2, 10]. For instance, increased coactivation of the trunk muscles and delayed response time after perturbation have been reported in people with cNLBP in the voluntary rapid arm flexion [2, 10]. The delayed muscle activation caused by APAs and CPAs dysfunction contributes to instability of the lumbar spine and the persistence of symptoms of low back pain [8]. The distal muscles of the lower limbs may also play a role in balance and postural control performance. The gastrocnemius (GA) muscle works across the knee and ankle joints, which indirectly influence the hamstring function [11]. This may subsequently alter the optimal pelvic rhythm during movement and place additional stress on of the spinal structure, contributing to tissue damage and pain [12]. Published study reported that GA dysfunction increased the risk of back injury in sportspersons [12], and fatigue of the GA was associated with a reduction of APAs timing variability in healthy individuals [13]. Recent published meta-analysis indicated the role of distal lower limb muscles of the ankle and knee joints in low back pain remains unclear [2].

Clinical symptoms of pain intensity reported to be associated with the magnitude of postural sway [14], and a reduction of pain intensity was associated with a smaller postural sway movement [15]. A potential explanation for the observed association was that pain may contribute to an increase in presynaptic inhibition of muscle afferents, leading to a decrease in muscle spindle feedback and prolonged latency in postural control. The prolonged latency in postural control may lead to additional load on the tissue structure and further pain. According to the evidence mentioned above, the majority of the current published studies focused in confirming the existence of APAs dysfunction and adopted clinical outcomes such as center of pressure or body sway trajectory to corroborate with clinical symptom. Some previous studies only explored the relationship between muscle onset and clinical symptoms of pain [9, 16]. However, the relationship between muscle activities during APAs/CPAs and clinical symptoms of pain remains unclear. Further understanding of such relationship would improve the modulation effect of postural adjustment on pain symptoms in people with CNLBP. Therefore, this study aimed to investigate the relationship between APAs\CPAs recorded during internal and external perturbation tasks, and their relationship between pain intensity and disability levels in patients with cNLBP. It was hypothesized that APAs dysfunction and CPAs dysfunction of the trunk and lower limb muscles were related to the clinical outcomes (including pain rating and disability) in people with cNLBP.

## 2. Methods

### 2.1. Sample Population

The sample population was recruited from the student population of the hosting institute and from the community. Advertisements were displayed around the university campus and circulated on social media platform. The diagnosis of LBP was made by a physiotherapist based on the clinical assessment protocol established by the American College of Physicians and American Pain Society [17]. The inclusion criteria for the cNLBP group were as follows: (1) age between 18 and 30; (2) been diagnosed with cNLBP for over 3 months; (3) pain score greater than 2 on the numerical rating scale (NRS) while at rest or during movement; (4) straight-leg raise test negative [18]; and (5) no clinical evidence of congenital anomalies of the lumbosacral region. The exclusion criteria for the cNLBP group were as follows: (1) presence of scoliosis as identified by Adam's forward bend test [19]; (2) history of fracture or surgery in the pelvic or spinal area; (3) history of a neurological disorder or on regular medications; and (4) pregnancy. Education level matched individuals were recruited for the control group. This study recruited young adults with cNLBP as a published study indicated the prevalence of LBP among the young adult population is on the rise [20]. Identifying the relationship between APAs/CPAs and pain level/disability may assist the early prevention or intervention of cNLBP [21].

### 2.2. Postural Control Tasks

**Figure 1:**
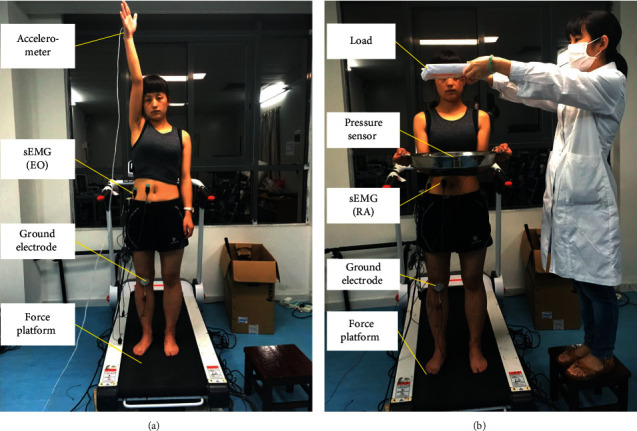
The experimental setup of the external and internal perturbation tasks. (a) Internal perturbation task. (b) External perturbation task.

In the present study, postural control tasks included external and internal perturbation tasks. During internal perturbation task (Figure 1(a)), participants stood on the force platform with their feet shoulder-width apart. The participant was asked to forward raise the dominant arm as fast as possible to approximately 180 degrees once the verbal cue “start” was given [9]. The verbal cue “start” was preceded by a verbal prompt “ready” for a random period of between three and four seconds. An accelerometer was attached to the wrist of the dominant arm to record the angular displacement of the dominant arm and act as a time marker [22]. During the external and internal perturbation tasks, the data of electrical activities of the four muscles on the dominant side, pressure sensor, accelerometer, and the center of pressure (COP) were recorded simultaneously.

In the external perturbation task (Figure 1(b)), participants were instructed to maintain an upright stance while standing on the force platform with their feet shoulder-width apart [23]. Previous study reported a load of 1.5 kg was sufficient to elicit APAs response, of which the intensity and onset time were similar to those in response to other load weights [23]. Thus, a 1.5 kg mass load was placed above the tray at the eye level to elicit APAs response. Each participant was asked to receive the load using a tray in their hands while maintaining a standing posture with the elbow kept at 90 degrees flexion. During this process, the participant was asked to gaze at the load and to maintain the stability of the body and the tray. A pressure sensor was positioned at the center of the tray to detect the pressure signals, which was the employed to determine the onset time of perturbation. Each participant completed three practice trials prior to testing. Five trials of perturbations were conducted with participants eyes opened. The duration of each trial was twenty seconds.

### 2.3. Instrumentation

#### 2.3.1. Surface Electromyography

Since the latencies of the trunk muscles on the ipsilateral side of arm movement in cNLBP participants were reported to be longer than those on the contralateral side during flexion and abduction [16], four muscles on the dominant side of the body: external oblique (EO), rectus abdominis (RA), erector spinae (ES), and GA were recorded by a four-channel surface electromyography (sEMG) system (Myomonitor IV, Delsys, USA) to obtain the APAs and CPAs capacity. The site for the electrode attachment of the EO was identified as the midpoint of the axial line between the 10th rib and the anterior superior iliac spine. The electrode attachment sites for RA and ES were identified as 3 cm lateral to the umbilicus and 3 cm lateral to the first lumbar vertebra, respectively. The one-third distance from the head of the fibula to the lateral side of the Achilles tendon insertion was marked as the electrode attachment site for the GA muscle [24]. A pair of surface electrodes with 25 mm apart was attached to the belly of each of the test muscles after the skin area was cleaned with alcohol wipes. A ground electrode was attached to the patella. sEMG signals were recorded with a gain of 1000 and amplified with differential amplifiers (Mega, Electronics Ltd, Finland).

#### 2.3.2. sEMG Signal Processing

**Figure 2:**
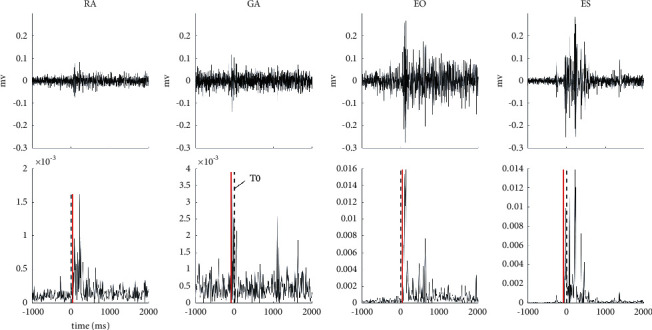
Illustrations of muscle onsets detected by the TKE operation. The upper panel is the raw sEMG data of the four muscles. The lower panel is the sEMG signals after the TKE operation. The black dashed lines are the onset time of the perturbation (T0); the red lines are the muscle onsets for each muscle. RA, rectus abdominis; GA, gastrocnemius; EO, lateral external oblique; ES, erector spinae.

Data processing was conducted offline in the software MATLAB (MathWorks, Natick, MA, USA). sEMG signals were full-wave rectified and band-pass filtered using a 2nd order, zero-lag Butterworth filter between 10 Hz and 500 Hz. In the internal perturbation task, the accelerometer data were averaged with a moving average filter of 10 seconds. The onset time of the internal perturbation task was defined as the time point where the acceleration magnitude reached 5% of the maximum of the square sum of the angular displacements in the medial-lateral and anterior-posterior directions. In the external perturbation task, the onset time of interruption (T0) was calculated from the pressure sensor when the pressure magnitude reached 5% of the maximum pressure for 10 ms. Each trial was aligned by T0 as a common reference point for sEMG signals. The sEMG data recorded between −600 ms (600 ms prior to T0) and  + 1000 ms (1000 ms post T0) of each trial was included for analysis. The onset time of each muscle could not be detected by the conventional method where sEMG amplitude was greater than the mean plus two standard deviation of the baseline value for at least 50 ms due to the presence of background noise. The Teager–Kaiser energy (TKE) operation is a computation algorithm that simultaneously considers the amplitude and instantaneous frequency of the sEMG when detecting muscle onset. It is applied in situation where the signal-to-noise ratio of the sEMG signals is too low to detect muscle onset by visual inspection of the signal or by setting a predetermined amplitude threshold [25]. The TKE operation was applied to all of the muscles in all of the trials. The detection time window was between −500 ms and +200 ms [25]. The computation algorithm of TKE operation adopted in the present study followed the procedure described the Li et al.'s study [25]. The muscle onsets of one trial detected by TKE operation are shown in Figure 2.

**Figure 3:**
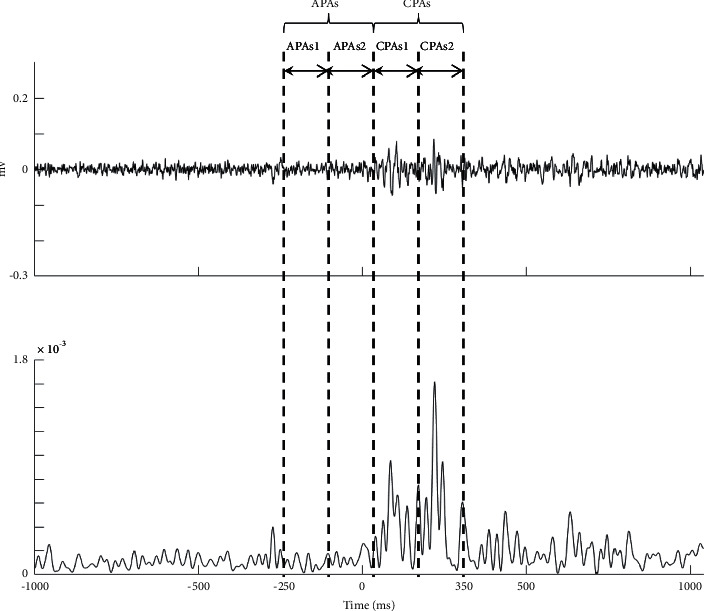
The time window of each subepoch during APAs and CPAs period. The upper panel is the raw sEMG data of rectus abdominis. The lower panel is the sEMG signals after the TKE operation. The zero point at the time axis is the onset time of perturbation.

Integrals of the EMG activities (iEMGs) of two different epochs were calculated for APAs and CPAs. In order to explore the dynamic change in the early and late APAs and CPAs, the time windows of APAs and CPAs in the present study were further divided into two subepochs. The time window of the epoch for APAs was from −250 ms to +50 ms, which could be divided into two subepochs: from −250 ms to −100 ms (APAs1) and from −100 ms to +50 ms (APAs2) (Figure 3). The time window of the epoch for CPAs was from +50 ms to +350 ms, which could be divided into two subepochs: from +50 ms to +200 ms (CPAs1) and from +200 ms to +350 ms (CPAs2) (Figure 3) [26]. The iEMGs for each subepoch were corrected by the iEMGs of the baseline activity from T0−600 ms to T0−450 ms, which was the same duration as each subepoch. However, the duration of iEMGs for APAs and CPAs was twice as long as the baseline duration and was corrected by doubling the iEMGs of baseline. Therefore, the formula adopted to calculate the iEMGs of muscles for APAs and CPAs was

(1) $$\begin{array}{c} i\text{EMGs}=\frac{\left(a-2b\right)}{2b}. \end{array}$$

where *a* is the iEMGs for APAs and CPAs and *b* is the iEMGs at baseline.

The formula adopted to calculate the muscle iEMGs during APAs1, APAs2, CPAs1, and CPAs2 was

(2) $$\begin{array}{c} i\text{EMGs}=\frac{\left(a-b\right)}{b}. \end{array}$$

where *a* is the iEMGs for each epoch of APAs1, APAs2, CPAs1, and CPAs2 and *b* is the iEMGs at baseline.

#### 2.3.3. Centre of Pressure

The force platform used to assess the COP displacement made reference to the study by Luo et al. [27]. The raw COP data were digitally filtered with fourth-order zero-lag Butterworth low-pass filter, of which cutoff frequency was set to 20 Hz. The COP displacements at T0 in medial-lateral and anterior-posterior directions were related to APAs, whereas the peak COP displacements (maximum displacement after T0) in *X* and *Y* axes were related to CPAs. The calculation details of COP displacement make reference to the previously published studies [24, 27, 28]. The COP displacements were also computed by MATLAB (MathWorks, Natick, MA, USA) program.

#### 2.3.4. Pain and Disability

Pain intensity was assessed by the 0–10 numerical rating scale (NRS) (Childs, 2005). Participants were asked to rate their pain intensity over the past 7 days prior to data collection. LBP-related disability was assessed by the Oswestry Disability Index (ODI) [29]. It consists of ten questions, and each question has six responses that correspond to a score between 1 and 5. The higher the score, the higher the disability level. A total score of 50 would indicate 100% disability [30, 31].

### 2.4. Experimental Procedure

Demographic data of age, height, weight, and body mass index (BMI) were recorded at the beginning of the data collection session, followed by the completion of clinical history questionnaire, NRS, and ODI. Then the external and internal perturbation tasks with signal recording of sEMG and COP were conducted. The order of the external and internal perturbation tasks was randomized for each participant.

### 2.5. Statistical Analysis

Descriptive statistical analysis was conducted to describe the sample characteristics. The independent-sample *t* test was performed to assess the difference in demographic characteristics except sex, muscle onset time, and muscle iEMGs between the two groups. The chi-square test was performed to assess the between-group difference in sex. The Pearson correlation analysis was performed to explore the relationship between the parameters of muscle onsets/iEMGs/COP and ODI scores. The Spearman correlation analysis was conducted to explore the relationship between the parameters of muscle onsets/iEMGs/COP and NRS ratings. Statistical analysis was performed in the software SPSS 20 (SPSS Inc., Chicago, USA). Statistical significance level was set at *p* < 0.05.

## 3. Results

### 3.1. Sample Populations

Twenty-seven participants (18 females and 9 males) with cNLBP were recruited. A further 27 healthy individuals (16 females and 11 males) were recruited as controls. Twenty-five of the cNLBP participants had bilateral low back pain or had pain at the central part of the lumbosacral area. Two of the cNLBP participants had unilateral low back pain at the lumbosacral area. All recruited participants were right-handed, with normal or corrected-to-normal visual acuity and no history of neurological and cardiovascular disorders. The demographic characteristics of the sample populations are shown in Table 1.

### 3.2. Muscle Onset, iEMGs, and COP in the Internal Perturbation Task

**Figure 4:**
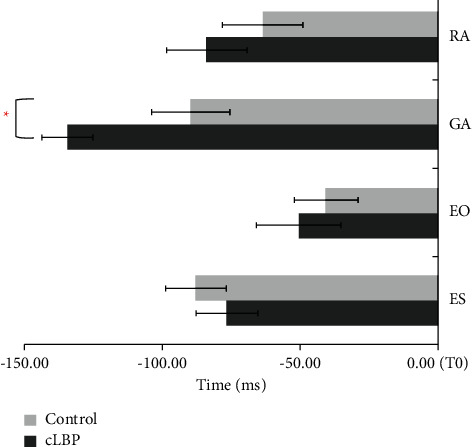
The onset time of muscle activity between the participants in the cNLBP and control groups in the internal perturbation task.

The onset time of muscle activities in internal perturbation task between the participants in the cNLBP and control groups is shown in Figure 2. The GA onset time was earlier in cNLBP patients (Figure 4), which suggested that cNLBP patients recruited GA earlier to keep balance before perturbation in the internal perturbation task. The muscle iEMGs in the internal perturbation task between the participants in the cNLBP and control groups are shown in Table 2. There were significant between-group differences in the iEMGs of RA during APAs (*p*=0.024) and APAs2 (*p*=0.016), the iEMGs of ES during APAs and APAs1 (*p*=0.029, *p*=0.024, respectively), and the iEMGs of ES during CPAs (*p* = 0.047). However, the iEMGs of GA and EO during APAs1 and APAs2 did not show significant between-group differences (Table 2).

Due to technical difficulties with the force platform during data collection, only 24 cNLBP participants and 16 control participants' COP data in internal perturbation task could be included in the data analysis. There were no significant differences in the COP displacement between two groups (*p* > 0.05) (Table 3).

### 3.3. Muscle Onset, iEMGs, and COP in the External Perturbation Task

**Figure 5:**
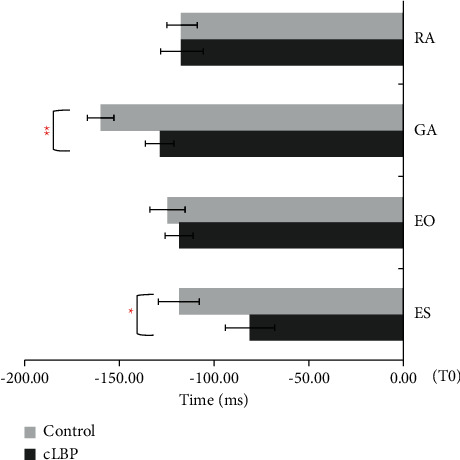
The muscle onset time between the cNLBP and control groups in the external perturbation task. RA, rectus abdominis; GA, gastrocnemius; EO, lateral external oblique; ES, erector spine. ^*∗*^*p* < 0.05; ^∗∗^*p* < 0.01.

Muscle onset time in the external perturbation task between the participants in the cNLBP and control groups is shown in Figure 4. The onset time of GA and ES of the control group was earlier than that of the cNLBP groups (Figure 5), which indicated that GA and ES were activated earlier to keep balance before perturbations in the control group during the external perturbation task. The iEMGs of GA during APAs and APAs1 were significantly lower in cNLBP participants than controls in the external perturbation task (*p*=0.029, *p*=0.046). However, the iEMGs of EO during APAs2 were larger in the cNLBP participants than controls (*p*=0.030) (Table 4).

In the external perturbation task, 23 cNLBP participants and 17 control participants' COP data were available for data analysis. Larger COP displacement in the *y* direction during CPAs was observed in the cNLBP group than the control group (*p*=0.010) (Table 5).

### 3.4. The Relationships between ODI/NRS and Muscle Onset Time/iEMGs in the Internal and External Perturbation Tasks

**Figure 6:**
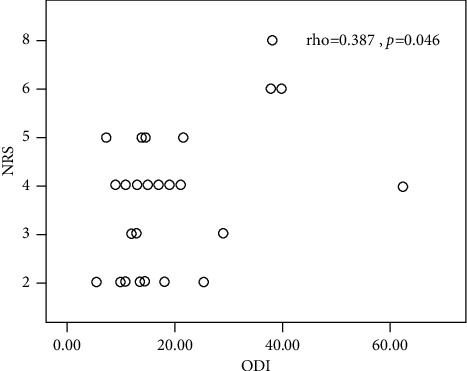
The association between ODI and NPR in cNLBP participants.

In the internal perturbation task, ODI scores of cNLBP participants were moderately correlated with the iEMGs of RA (during APAs: *r* = 0.425 *p*=0.027; during APAs2: *r* = 0.459, *p*=0.016) and GA (during APAs: *r* = 0.388, *p*=0.045; during APAs1: *r* = 0.506, *p*=0.007) (Table 6). ODI scores of cNLBP participants were correlated with the iEMGs of EO during APAs2 (*r* = 0.430, *p*=0.025). The NRS scores of cNLBP participants were moderately related to iEMGs of RA (during CPAs: *rho* = 0.444, *p*=0.020; during CPAs2: *rho* = 0.442, *p*=0.021), EO (during CPAs: *rho* = 0.402, *p*=0.038), and ES (during CPAs: *rho* = 0.437, *p*=0.023; during CPAs2: *rho* = 0.470, *p*=0.013) (Table 7). ODI was positively related to the NRS (rho = 0.387, *p*=0.046).  Figure 6 illustrates the relationship between ODI and NPR in cNLBP participants. No significant correlation between ODI scores and COP data and between NRS and COP data was observed in the internal perturbation task. No significant correlation between ODI scores and muscle onset time/iEMGs/COP data and between NRS scores and muscle onset time/iEMGs/COP data during APAs and CPAs in the external perturbation task (*p*>0.05).

## 4. Discussion

The present study investigated the relationship between postural control and clinical symptoms in the patients with cNLBP. The main findings showed that the activation of GA was earlier in the cNLBP group than the control group in the internal perturbation task. The activation of the muscles GA and ES of cNLBP participants were delayed when comparing to healthy controls in the external perturbation task. The activation of the trunk muscles during APAs and CPAs in the cNLBP group was stronger than those in the control group in the internal perturbation task. However, the muscle activation of the EO was stronger in the cNLBP group than the control group, whereas the muscle activation of the GA in the cNLBP group was weaker than those in the control group during the external perturbation task. The ODI scores of the cNLBP group were moderately correlated with the iEMGs of RA, GA, and EO during APAs1 or APAs2. However, the NRS scores of the cNLBP group were moderately related to the iEMGs of RA, EO, and ES during CPAs and CPAs2. These observed relationships are some of the novel findings of the present study, suggesting that pain-related disabilities of cNLBP participants were likely related to APAs capacity, whereas the pain intensity seemed to be related to CPAs capacity.

### 4.1. Muscle Onset Time

GA was activated earlier in the cNLBP group than the control group in the internal perturbation task. The direct comparison with published studies on GA activation was not feasible as the majority of the published studies that concerned APAs/CPAs impairments recorded activity of other muscle groups [2, 8, 9]. Most of the published studies reported a delay in the activation of transversus abdominis or transversus abdominis/internal oblique muscles, which are the major muscles to maintain postural stability, during rapid arm movement [2]. The sEMG signals of the transversus abdominis were not recorded in the present study as low signal-to-noise ratio was observed in our pilot study. In future study, the sEMG of transversus abdominis should be recorded to enable direct comparison with published study. No between-group difference in muscle onset of the EO was observed in the external perturbation task. This finding is consistent with previous studies [9, 32], which reported significant between-group difference in muscle onset time of the EO on the contralateral side of the arm movement, but no significant difference on the ipsilateral EO. EO muscle is the primary muscle to produce torque proportional to the direction [8], whereas contralateral EO might play a greater role in controlling trunk stiffness. Thus, future study should explore the interaction of the bilateral postural muscles during perturbation tasks.

In the external perturbation task, the onset times of GA and ES of cNLBP participants were delayed comparing to the healthy controls. These are consistent with the findings of previous studies where a delayed activation of trunk or lower limb muscles in response to external perturbation [2, 33] was observed in cNLBP participants. The delay in muscle activation pattern to restore postural ability was different during external and internal perturbation tasks. The perturbation in the internal task is endogenous where participants could have adequate preparation period before the initiation of motion. However, the perturbation in the external task is exogenous. Thus, cNLBP participants might not have sufficient time to generate APAs response since the time for the load to drop on the plate is very short. This finding further supports the APAs dysfunction in patients with cNLBP.

### 4.2. Muscle iEMGs

The iEMGs of the trunk muscles RA and ES during APAs phase were larger in participants with cNLBP than healthy participants during the internal perturbation task. These findings suggested higher trunk muscle activation to stabilize the spine. These findings are consistent with those reported by Ringheim et al. [34]. The findings in Ringheim et al.'s study showed that patients with cNLBP required increased level of muscle activation to minimize the postural perturbation. RA-ES is one of the important muscle pairs in postural control [26]. They work synergistically and assist in the anticipatory shifts of COP to achieve postural stability [35]. The deep core muscles in the people with CLBP are commonly reported to be weak. It is therefore likely that the increased activation of superficial core muscles (e.g., RA, ES) and earlier muscle activation (e.g., ES) were compensation for the weak deep muscles to increase trunk stiffness to stabilize the spine [36, 37]. Thus, cNLBP participants in the present study increased the superficial muscle activation of RA-ES in response to internal perturbation to achieve postural stability. In the present study, the iEMGs of RA-ES during theAPAs2 phase were larger than those during APAs1 phase in both groups. These findings are consistent with studies that reported the largest anticipatory responses during the APA2 epoch [7, 26], suggesting the CNS could generate APAs in a time point that is close to the moment of the perturbation occurrence. However, the iEMGs of the ES during APAs1 phase in the cNLBP group was larger than the control group. The might relate to the trunk muscle coordination impairment in cNLBP [38], so that an earlier and stronger contraction of trunk muscles is required to maintain balance.

In the external perturbation task, the iEMGs of the EO were larger in the cNLBP group than the control group, whereas the iEMGs of GA in the cNLBP group were smaller than those in the control group. These findings may indicate altered postural muscle coordination in patients with cNLBP. Several published studies previously reported altered muscle coordination of the trunk and lower limbs in the patients with cNLBP [36, 39]. It is common that people depend more on the ventral muscles of the lower limb (e.g., tibialis anterior) rather than the dorsal muscles (e.g., GA) in response to the predictable external perturbation [26]. Santos and Aruin [35] found a reciprocal activation strategy of ventral and dorsal muscles when the task was relatively easy, but a coactivation of ventral and dorsal muscles when the task was more challenging. cNLBP patients activated tibialis anterior (ventral muscle) than the healthy controls in the response to the external perturbation, which was not observed in GA (dorsal muscle) of cNLBP patients [33]. In the present study, the cNLBP patients may recruit more of the ventral muscles (e.g., EO) rather than the dorsal muscles (e.g., GA) to maintain stability for the predictable external perturbation since the task with mass of perturbation load was not challenging. This is a potential reason that the iEMGs of EO and GA were different between thecNLBP and control groups. However, the muscle activation of tibialis anterior was not detected in the present study. Thus, no definitive conclusion could be drawn from this as no consistent pattern between all of the ventral and dorsal muscle was observed in the present study. Future study is required to substantiate the findings of the present study. In addition, despite the iEMGs of GA recorded in the cNLBP group were negative, the activation of GA was weak and the values were close to zero. Thus, it could not be concluded that the GA was inhibited during both APAs and CPAs. The iEMG of the GA in the cNLBP group was significantly different to the control group. This is consistent with the finding reported by Hemmati et al. [33] where a lower muscle activation of GA was observed in the cNLBP group than the control group. This provides further evidence of altered muscle activation pattern in cNLBP patients.

Due to technical difficulties with the force platform, the COP data from 14 participants collapsed during data collection of the internal and external perturbation tasks. This is a possible reason that affect the robustness of the analysis of between-group difference and relationship in COP data, which would not be discussed in this study.

### 4.3. Correlations of Muscle Onset Time, Pain, and Disability in the Internal Perturbation Task

All of the onset time of ipsilateral muscles relative to the arm movement was not significantly related to the pain rating and ODI. There were several studies to explore the relationship among muscle onset time, pain, and disability. One study reported a positive correlation between contralateral EO onset and ODI and physical scores of the Fear Avoidance Beliefs Questionnaire (FABQ) [9]. However, another study found a significant positive correlation between the onset time of IO muscle and pain severity [16]. Thus, the relationship among muscle onset time, pain, and disability may be muscle dependent. Future study should detect more muscles to explore the associations among muscle onset time/iEMGs, pain, and disability.

### 4.4. Correlations between Muscle IEMGs and Disability/Pain in the Internal Perturbation Task

ODI was moderately correlated with the iEMGs of GA in the early phase of APAs (APAs1) and the iEMGs of RA and EO in the late phase of APAs (APAs2). These suggested that the iEMGs of GA, RA, and EO for APAs were related to the disability in our daily tasks. The GA is the agonist muscle for knee flexion which indirectly affected the hamstrings and limits the range of trunk forward bending [12, 40]. It was reported that cNLBP patients had high muscle tension of the GA [40], and stretching intervention of the GA might reduce pain and improve functional level in patients with cLBP [31]. These studies supported that the iEMGs of GA were related to the disability performance in our daily tasks. The activation of GA at the early phase of APAs (APAs1) and the RA and EO at the late phase of APAs (APAs2) are to maintain balance for the up and forward torque at the beginning of the rapid arm lifting task [41] via a dorsal-to-ventral muscle activation sequence. The patients with cNLBP might adopt an alternative strategy by activating the distal muscles in the early phase of APAs due to the weak deep core muscles. However, the difference of iEMGs between the cNLBP and control group was only observed in the RA, rather than GA, during late APA phase in the internal perturbation task. This suggested that during APAs, RA was likely to contribute more than GA to postural instability in the internal perturbation.

The NRS scores of cNLBP participants were moderately associated with the iEMGs of RA, EO, and ES during CPAs and CPAs2. This finding indicated that the patients with higher pain intensity demonstrated larger muscle activation of postural muscles during CPAs. Our findings were supported by the findings of another study [14], which also reported positive association between pain intensity and postural sway in people with cNLBP. Pain may cause an increased presynaptic inhibition of muscle afferents [42], which in turn contributes to a high-threshold nociceptive afferents at the low back that interferes with spinal motor pathways and the motor cortex. Pain interference also affected the central modulation of proprioceptive spindles of muscles [43], leading to higher iEMGs of superficial core muscle (e.g., ES) for the imperative perturbation in the patient with cNLBP.

### 4.5. Correlations between Muscle Onset/IEMGs and Disability/Pain in the External Perturbation Task

This study found no significant correlations between ODI/NRS and muscle onset/iEMGs during APAs and CPAs in the external perturbation task. The potential reason was the chosen perturbation load of 1.5 kg might be insufficient to fully activate the muscle fibers during CPAs, as it was lighter than 5% of participants that was adopted in a previous study [44]. Accordingly, it should take caution to make a conclusion for the association between ODI/NRS and muscle iEMGs in the external perturbation task. There were still lack of studies exploring the relationship between muscle activities during APAs/CPAs and clinical symptoms of pain in the external perturbation task.

## 5. Limitations

There are several limitations in the present study. First, the weight of the load in the external perturbation task was only 1.5 kg, which did not reach 5% of participant's weight used in the previous study and could not elicit strong muscle activation during CPAs. Thus, the between-group differences and the relationship between postural control and pain-related outcomes in the external perturbation task could not be found. In the future study, the weight of the external loading should reach 5% of participant's weight. Second, the age range of sample population is from 21 to 27. The findings in the present study may not be accurately generalized to other age ranges of population. Third, the present study did not consider the difference of muscle activation pattern between the painful and nonpainful sides or between the contralateral and ipsilateral sides of the arm movement, which may prevent further conclusion to be drawn on the muscle activation pattern of cNLBP patients. Fourth, even though the between-group differences were observed in the present study, the findings only could reflect the general capacity of postural control in the population assessed. Some of the included cNLBP participants had comparable postural control ability as some of the participants in the control group. The variability between the two groups was different in some outcome variables according to the standard deviations, which may reduce the generalizability of the data. Future study with larger sample size to enable subgroup analysis based on postural control impairment is recommended. Fifth, sample calculation was not conducted in the present study. The underpowered sample size might lead to Type-II error.

## 6. Conclusion

People with cNLBP adopted an altered strategy by increasing the activation of the superficial trunk muscles and dorsal muscles in APAs to achieve postural stability in response to internal perturbation. Different muscle activation pattern in internal and external perturbation tasks of cNLBP group suggested that the muscle activation in APAs and CPAs was task dependent. Pain-related disabilities of cNLBP participants are likely related to the APAs capacity, whereas the pain intensity may relate to the CPAs capacity. Pain and disability may therefore be related to the APAs/CPAs control process of the posture-related muscles.

## Figures and Tables

**Table 1:** The demographic characteristics of the sample populations.

	cNLBP (mean ± SD, range)	Control (mean ± SD, range)	*t*	*p*
Age (years)	23.77 ± 3.42, 11.00	22.67 ± 1.98, 11.00	1.460	0.150
Sex (male/female)^a^	18/9	16/11	N.A.	0.573
Height (m)	165.96 ± 8.15, 33.00	166.33 ± 8.10, 29.00	−0.167	0.868
Weight (kg)	58.33 ± 11.13, 45.00	56.56 ± 8.82, 36.00	0.648	0.520
BMI (kg/m^2^)	21.07 ± 3.01, 11.51	20.39 ± 2.34, 9.94	0.939	0.352
ODI	18.94 ± 12.61, 56.80	NA		
NRS	3.67 ± 1.57, 6	NA		
Pain duration (months)	20.41 ± 20.41, 69			

Notes: ^*a*^chi-square test was conducted to assess the difference in sex. BMI, body mass index; ODI, Oswestry Disability Index; NRS, Numerical Rating Scale.

**Table 2:** The muscle iEMGs between the cNLBP and control groups in the internal perturbation task.

	cNLBP (mean ± SD)	Control (mean ± SD)	*t*	*p*
APAs_RA_	0.253 ± 0.369	0.077 ± 0.110	2.371	0.024^*∗*^
APAs1_RA_	0.157 ± 0.321	0.049 ± 0.104	1.664	0.106
APAs2_RA_	0.357 ± 0.471	0.113 ± 0.159	2.550	0.016^*∗*^
CPAs_RA_	1.144 ± 1.306	0.760 ± 1.063	1.184	0.242
CPAs1_RA_	0.760 ± 1.014	0.402 ± 0.471	1.668	0.104
CPAs2_RA_	1.540 ± 1.773	1.131 ± 1.733	0.857	0.395
APAs_GA_	0.087 ± 0.168	0.160 ± 0.390	−0.892	0.376
APAs1_GA_	0.062 ± 0.128	0.110 ± 0.341	−0.682	0.498
APAs2_GA_	0.119 ± 0.246	0.271 ± 0.558	−0.841	0.404
CPAs_GA_	0.504 ± 0.650	0.484 ± 0.351	0.142	0.887
CPAs1_GA_	0.474 ± 0.709	0.530 ± 0.512	−0.330	0.743
CPAs2_GA_	0.547 ± 0.735	0.450 ± 0.458	0.582	0.563
APAs_EO_	0.455 ± 0.678	0.308 ± 0.544	0.881	0.383
APAs1_EO_	0.351 ± 0.880	0.186 ± 0.357	0.899	0.373
APAs2_EO_	0.571 ± 0.668	0.436 ± 0.786	0.677	0.501
CPAs_EO_	2.374 ± 2.393	1.887 ± 2.118	0.807	0.423
CPAs1_EO_	1.403 ± 1.386	1.065 ± 0.810	1.096	0.279
CPAs2_EO_	3.364 ± 3.689	2.713 ± 3.709	0.646	0.521
APAs_ES_	0.857 ± 1.137	0.336 ± 0.331	2.288	0.029^*∗*^
APAs1_ES_	0.611 ± 0.964	0.154 ± 0.253	2.385	0.024^*∗*^
APAs2_ES_	1.119 ± 1.480	0.526 ± 0.558	1.947	0.060
CPAs_ES_	3.049 ± 2.723	1.943 ± 1.559	1.830	0.074
CPAs1_ES_	2.116 ± 2.085	1.882 ± 1.743	0.448	0.656
CPAs2_ES_	4.004 ± 4.242	2.155 ± 1.937	2.060	0.047^*∗*^

RA, rectus abdominis; GA, gastrocnemius; EO, lateral external oblique; ES, erector spinae. ^*∗*^*p* < 0.05.

**Table 3:** The COP displacement between the cNLBP and control groups in the internal perturbation task.

	cNLBP (*n* = 24) (mean ± SD)	Control (*n* = 16) (mean ± SD)	*t*	*p*
X-COP at T0	8.65 ± 5.82	13.29 ± 22.64	−0.802	0.434
Y-COP at T0	6.38 ± 4.14	8.00 ± 11.55	−0.629	0.533
X-COP at peak	26.87 ± 17.15	37.23 ± 49.62	−0.804	0.432
Y-COP at peak	24.33 ± 22.19	29.37 ± 24.32	−0.678	0.502

X-COP at T0, the displacement of the center of pressure (COP) in *x* direction during APAs; Y-COP at T0, the displacement of COP in *y* direction during APAs; X-COP at peak, the peak displacement of COP in *x* direction during CPAs; Y-COP at peak, the peak displacement of COP in *y* direction during CPAs.

**Table 4:** The muscle iEMGs between the cNLBP and control groups in the external perturbation task.

	cNLBP (mean ± SD)	Control (mean ± SD)	*t*	*p*
APAs_RA_	0.017 ± 0.114	−0.019 ± 0.087	1.296	0.201
APAs1_RA_	0.019 ± 0.167	−0.022 ± 0.121	1.029	0.308
APAs2_RA_	0.022 ± 0.113	−0.009 ± 0.088	1.121	0.268
CPAs_RA_	0.031 ± 0.119	0.000 ± 0.084	1.103	0.275
CPAs1_RA_	0.033 ± 0.132	0.003 ± 0.197	0.971	0.336
CPAs2_RA_	0.037 ± 0.138	0.004 ± 0.095	1.009	0.318
APAs_GA_	−0.006 ± 0.064	0.030 ± 0.053	−2.244	0.029^*∗*^
APAs1_GA_	−0.011 ± 0.082	0.029 ± 0.061	−2.041	0.046^*∗*^
APAs2_GA_	0.005 ± 0.074	0.036 ± 0.074	−1.572	0.122
CPAs_GA_	−0.007 ± 0.068	0.012 ± 0.071	−0.965	0.339
CPAs1_GA_	−0.003 ± 0.077	0.022 ± 0.101	−1.014	0.315
CPAs2_GA_	−0.003 ± 0.078	0.008 ± 0.064	−0.571	0.571
APAs_EO_	0.065 ± 0.137	0.007 ± 0.142	1.535	0.131
APAs1_EO_	0.070 ± 0.212	0.032 ± 0.201	0.675	0.503
APAs2_EO_	0.067 ± 0.137	-0.011 ± 0.118	2.237	0.030^*∗*^
CPAs_EO_	0.074 ± 0.157	0.010 ± 0.139	1.581	0.120
CPAs1_EO_	0.081 ± 0.179	0.000 ± 0.126	1.934	0.059
CPAs2_EO_	0.073 ± 0.185	0.026 ± 0.195	0.912	0.366
APAs_ES_	−0.002 ± 0.144	−0.012 ± 0.104	0.296	0.769
APAs1_ES_	0.017 ± 0.146	−0.007 ± 0.120	0.665	0.509
APAs2_ES_	−0.013 ± 0.180	−0.010 ± 0.120	−0.079	0.938
CPAs_ES_	0.022 ± 0.113	0.002 ± 0.106	0.642	0.524
CPAs1_ES_	0.009 ± 0.137	0.001 ± 0.132	0.220	0.827
CPAs2_ES_	0.041 ± 0.118	0.010 ± 0.117	0.965	0.339

RA, rectus abdominis; GA, gastrocnemius; EO, lateral external oblique; ES, erector spine. ^*∗*^*p* < 0.05.

**Table 5:** The COP measurements of the cNLBP and control groups in the external perturbation task.

	cNLBP (mean ± SD)	Control (mean ± SD)	*t*	*p*
X-COP at T0	9.05 ± 7.35	5.96 ± 4.32	1.546	0.130
Y-COP at T0	6.22 ± 3.62	4.15 ± 3.97	1.714	0.095
X-COP at peak	26.45 ± 23.08	14.52 ± 12.22	1.936	0.060
Y-COP at peak	18.82 ± 10.64	10.75 ± 7.02	2.717	0.010

**Table 6:** Pearson correlation coefficient between ODI scores and muscle onset time/iEMGs in the internal perturbation task.

	RA	GA	EO	ES
Onset time-ODI	−0.087	−0.327	−0.009	0.014
APAs-ODI	0.425^*∗*^	0.388^*∗*^	0.381	0.249
APAs1-ODI	0.309	0.506^∗∗^	0.263	0.248
APAs2-ODI	0.459^*∗*^	0.265	0.430^*∗*^	0.221
CPAs-ODI	0.134	−0.122	0.103	0.104
CPAs1-ODI	0.220	−0.101	0.215	0.171
CPAs2-ODI	0.072	−0.118	0.054	0.050

^*∗*^ *p*<0.05; ^∗∗^*p*<0.01.

**Table 7:** Spearman's correlation analysis between NRS scores and muscle onset time/iEMGs in the internal perturbation task.

	RA	GA	EO	ES
Onset time-NRS	−0.017	−0.106	−0.078	−0.095
APAs-NRS	0.264	−0.040	0.297	0.121
APAs1-NRS	0.126	0.046	0.145	0.044
APAs2-NRS	0.368	−0.106	0.325	0.161
CPAs-NRS	0.444^*∗*^	0.262	0.402^*∗*^	0.437^*∗*^
CPAs1-NRS	0.363	0.021	0.341	0.136
CPAs2-NRS	0.442^*∗*^	0.361	0.361	0.470^*∗*^

^*∗*^ *p*<0.05.

## Data Availability

Data are available upon request from the corresponding authors.
